# Uncovering genetic population structure in the Endangered northern rockhopper penguin (*Eudyptes moseleyi)* across islands in the Southern Atlantic and Indian oceans

**DOI:** 10.1186/s12864-025-12487-9

**Published:** 2026-01-06

**Authors:** Heather Ritchie-Parker, Alex Ball, Trevor Glass, Jean-Marc Costanzi, Thierry Boulinier, Amandine Gamble, Muhammad Ghazali, Karen Keegan, Christophe Barbraud, Charly Bost, Caroline Bost, Jaimie Cleeland, Augustin Clessin, Maelle Connan, Karine Delord, Ben Dilley, Tristan Glass, Christopher W. Jones, Peta Moore, Kate Lawrence, Fabrice LeBouard, Alexis Osborne, Richard A. Phillips, Norman Ratcliffe, Julian Repetto, Michelle Risi, Peter G. Ryan, Brandon Spolander, Shannon Swain, Wayne Swain, Jérémy Tornos, Alex Whittle, Helen Senn, Antje Steinfurth

**Affiliations:** 1 RZSS WildGenes, Conservation Department, Royal Zoological Society of Scotland, Edinburgh, UK; 2 Tristan da Cunha Government, Conservation, Edinburgh of the Seven Seas, Tristan da Cunha, United Kingdom; 3 CEFE, CNRS, Université Montpellier, EPHE, IRD, Montpellier, France; 4 Centre d’Etudes Biologiques de Chizé (CEBC), UMR 7372CNRS-La Rochelle Université, Villiers-en-Bois, France; 5 British Antarctic Survey, Cambridge, UK; 6 Department of Zoology, Marine Apex Predator Research Unit, Institute for Coastal and Marine Research, Nelson Mandela University, Gqeberha, South Africa; 7 FitzPatrick Institute of African Ornithology, University of Cape Town, Cape Town, South Africa; 8 Centre for Conservation Science, The Royal Society for the Protection of Birds, Cambridge, UK; 9 Perth Zoo, South Perth, Western Australia; 10 Two Oceans Aquarium, Cape Town, South Africa; 11 Cambridge Conservation Initiative, The Royal Society for the Protection of Birds, Cambridge, UK

**Keywords:** Penguin, Conservation, Connectivity, Gene flow, Migration, Management, ddRAD, mtDNA

## Abstract

**Background:**

The northern rockhopper penguin (*Eudyptes moseleyi*) is a threatened species, listed as Endangered globally by the IUCN owing to rapid population decreases in the past, combined with a limited distributional range. Their breeding is confined to five islands in the central South Atlantic Ocean (Gough, Nightingale, Inaccessible, Alex (= Middle), and Tristan da Cunha) and two islands in the southern Indian Ocean (Amsterdam and Saint Paul). Non-breeding birds forage widely in these oceans and vagrant individuals have been recorded in the Falkland Islands/Malvinas, South Africa, Kerguelen Archipelago, Australia, and New Zealand. The origins of these vagrant birds are largely unknown, and it remains unclear to what extent northern rockhopper penguins move between islands and oceans. Understanding connectivity between populations is essential for developing appropriate conservation strategies, especially as some populations may be at greater extinction risk than others.

**Results:**

Northern rockhopper penguins from the two oceanic basins are genetically distinct, with minimal evidence of migration between ocean basins. No substructure was detected within ocean island groups and although low, the level of migration between islands was sufficient to prevent genetic differentiation. Differential signatures of genetic diversity and inbreeding may also suggest some island populations are at higher risk of inbreeding depression.

**Conclusions:**

This study provides the first comprehensive genetic population structure analysis of the entire breeding range of the Endangered northern rockhopper penguin. This gives an unparalleled understanding of the connectivity within and between populations breeding in the Atlantic and Indian oceans, and highlights areas for further investigation. Knowledge of genetic structure and population dynamics can inform effective conservation management by predicting a species’ ability to adapt and remain resilient to local or global threats, including the increasing impacts of climate-driven changes in marine environments. Our results suggest that northern rockhopper penguins should be managed as two conservation management units to maximise the conservation of genetic diversity within the species and allow strategies to be developed that consider the different pressures affecting the populations in each ocean basin.

**Supplementary Information:**

The online version contains supplementary material available at 10.1186/s12864-025-12487-9.

## Background

Northern rockhopper penguins (*Eudyptes moseleyi*) are classified as Endangered on the IUCN Red List [1] due to extensive population declines over the last century, including a > 50% decline in the Atlantic population between 1975 and 2005 [2]. The species breeds on seven islands; five in the Tristan da Cunha island group in the central South Atlantic Ocean and two in the Indian Ocean, with vagrants also reported in the Falkland Islands/Malvinas [3, 4], Kerguelen Archipelago [5], Australia [6], New Zealand [7] and South Africa [8]. The last population assessment suggests a global total of around 200,000 breeding pairs remain in the wild [1]. Most individuals breed in the Atlantic (~ 90%) and population sizes vary from ~ 3600 pairs on Tristan da Cunha (hereafter Tristan) to ~ 64,700 pairs on Gough Island [1]. Population trends vary between islands with Saint Paul showing an increase [9, 10], Amsterdam and Gough showing decreases [2, 9–11], and Alex (= Middle), Inaccessible, Nightingale and Tristan remaining largely stable [12].

Like many other seabirds, northern rockhopper penguins are threatened by changes in their marine environment but the exact causes of their population decline are not fully understood [1] and the reasons for their decline may be island-specific and influenced by local factors. Broadly, they are thought to be affected by the consequences of climate change including changes in sea surface temperatures causing the reduction and displacement of prey, as well as possible depredation by growing populations of fur seals (*Arctocephalus tropicalis*) [1, 13–15]. Some populations have been affected by catastrophic events such as the 2011 incident where the bulk carrier MS Oliva ran aground on Nightingale Island resulting in the spillage of 300,000 gallons of heavy fuel oil which affected penguins on Nightingale, Alex and Inaccessible Islands [16] and resulted in the deaths of thousands of penguins [17]. Population declines on Amsterdam Island have been attributed to recurrent outbreaks of avian cholera, with the bacterium *Pasteurella multocida* posited to be responsible for low breeding success and complete breeding failure between 2013 and 2016 [18]. A more recent survey on Amsterdam Island revealed an accelerated decline in the population, likely caused by a major fire at one of the primary breeding sites [11].

Conservation management of the northern rockhopper penguin requires these threats to be better understood and addressed. Direct action has been taken to remove feral cattle (*Bos taurus*) on Amsterdam that may have indirectly affected the habitat due to grazing [19] as well as the eradication of predatory invasive species such as feral cats (*Felis catus*), brown rats (*Rattus norvegicus*) and house mice (*Mus musculus*) on Amsterdam Island [20]. Across all islands the birds are protected by law where populations in the Indian Ocean have been encompassed within the Réserve Naturelle Nationale des Terres Australes Françaises since 2006 [21, 22], and important foraging areas for northern rockhopper penguins in the Atlantic Ocean are embedded in the Tristan da Cunha Marine Protection Zone that was designated in 2020 [23]. Management plans have been implemented for many of the islands which include actions related to population monitoring, mitigation of the impact of human activities, and improving knowledge of the species to facilitate more effective management [24, 25]. In 2017, a species action plan was published to guide the ongoing conservation management of the species [26].

The northern rockhopper species action plan details key challenges in the conservation management of the species and outlines goals and objectives required for their recovery. A key objective is to fully understand the ecology and life history of northern rockhopper penguins by examining the causes of breeding failure, developing a better understanding of their diet and trophic ecology, and investigating connectivity between islands [26]. Connectivity is especially important in disparately located populations as it can be challenging to delineate management units [27]. Specifying suitable management units is particularly crucial when there is a source-sink dynamic between populations, as resources directed towards a source population will have a higher impact than those directed towards a sink population [28]. Understanding connectivity is also pertinent for identifying the risk of disease transfer between populations [29].

Previous genetic studies investigating the connectivity between populations of northern rockhopper penguins have been limited. The majority of these studies have focused on one island population in the Atlantic Ocean and one in the Indian Ocean. Studies that utilised nuclear data have shown low but significant levels of differentiation between birds on Amsterdam in the Indian Ocean and both Gough [30] and Nightingale [31] in the Atlantic Ocean. An investigation of connectivity between Amsterdam and Nightingale using a small number of mitochondrial and nuclear loci produced conflicting results with one mitochondrial marker showing differentiation between the populations, but one nuclear marker showed no such differentiation [32]. The single study of connectivity between island populations in the same ocean showed no differentiation between birds on Gough and Nightingale in the Atlantic Ocean based on one mitochondrial and six nuclear markers [33]. No study to date has examined connectivity across the entire species’ range with either mitochondrial or nuclear data.

Here we use molecular techniques to gain a better understanding of connectivity between island populations of northern rockhopper penguins to better advise on effective conservation management of the species. We utilised both mitochondrial markers (mtDNA) and double-digest Restriction site-associated DNA (ddRAD) to investigate connectivity between the islands of Tristan da Cunha (Alex, Gough, Inaccessible, Nightingale, and Tristan) in the central South Atlantic Ocean, and the islands of Amsterdam and Saint Paul in the southern Indian Ocean. Using these data, we (1) investigate patterns of gene flow within and between the islands in the Atlantic and Indian oceans, (2) estimate the extent and direction of migration between the island populations, and (3) determine the origin of vagrant individuals from western Australia and South Africa.

## Methods

### Sample collection and DNA extraction

**Fig. 1:**
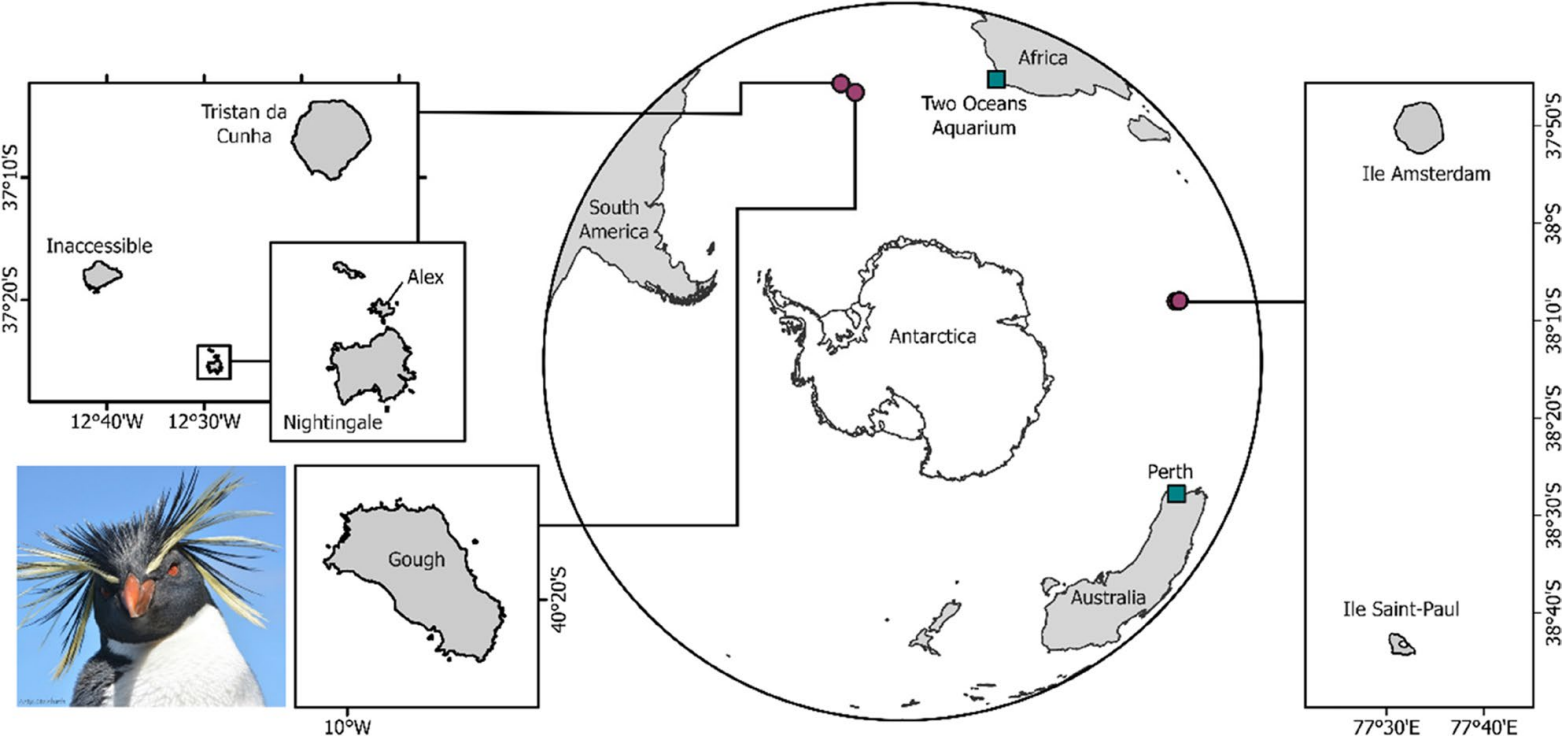
Map of sampling locations of northern rockhopper penguins. Red circles indicate penguins sampled at breeding colonies in the South Atlantic and southern Indian Ocean. Green squares indicate vagrants sampled from South Africa and western Australia. Insert photo shows a northern rockhopper penguin.

A total of 181 northern rockhopper penguins were sampled at five islands in the central South Atlantic (*n* = 125) and two islands in the southern Indian oceans (*n* = 56). Other birds sampled were a single vagrant found off the coast of Perth, western Australia, and 12 penguins housed at the Two Oceans Aquarium in Cape Town, South Africa which were vagrants to South Africa or their captive-bred descendants. Full details of sample locations are available in Fig. 1 and Supplementary Table 1.

Blood samples were stored in either EDTA, ethanol, or fixed onto FTA cards. In all cases, samples were extracted using a QIAGEN© DNeasy Blood and Tissue kit following the manufacturer’s standard protocol. DNA quantity was assessed using a Qubit 2.0 fluorometer and DNA quality was examined for the presence of high molecular weight DNA via electrophoresis on a 1% agarose gel.

## mtDNA sequencing and analysis

Samples were sequenced at two regions, a section of the mtDNA control region (CR) using the RockCR_F (5′-TGG CTT TTC TCC AAG ACC TG-3′) and RockCR_R (5′-TGG CTC TGT GAA GAG CAA GA-3′) primers [32] and a portion of the NADH dehydrogenase 2 (ND2) gene using the primers PEN_ND2_L5216_F (5′- GCC CAT ACC CCR AMA ATG-3′) and PEN_ND2_H5766_R (5′- GGA TGA GAA GGC TAG GAT TTT KCG-3′)[31] following the methods described by Frugone et al. (2018) [32] and Sorenson et al. (1999) [34], respectively. PCR products were visualised on a 1.5% agarose gel and purified using exoFAP before sequencing in both directions on an AB3130 XL sequencer (Eurofins, Germany).

Sequence quality was checked visually, primer sequences were trimmed, and sequence reads were assembled using Geneious Prime version 2025.0.1. Consensus sequences were aligned using the default parameters in MAFFT version 7.450 [35]. The resulting sequences were then compared to determine the haplotype identity of each sample. Haplotype networks were constructed in R using the ‘pegas’ package [36] for the control region only, ND2 only, and a combined dataset.

## ddRAD sequencing

A total of 134 individuals were prepared for ddRAD sequencing across two ddRAD libraries, each containing up to 96 individually barcoded samples. Inter- and intra-plate positive controls were used to check for sequencing quality and batch effects. Libraries were constructed using the method outlined by Peterson et al. (2012) [37], with slight adjustments as described in Dicks et al. (2023) [38], using SphI and SbfI restriction enzymes. Each sample was barcoded using a 5–7 bp barcode before being pooled and size-selected (400–700 bp). Libraries were sequenced as 150 bp paired-end reads on an Illumina HiSeq 4000 system.

Sequenced libraries were demultiplexed in STACKS version 2.52 [39] using *process_radtags*. Samples with fewer than 250,000 reads were removed due to insufficient coverage. Read adapters were removed by trimming reads to 135 bp in length, and low-quality reads were removed using default parameters. Retained reads were quality checked using FastQC [40] before being aligned to the northern rockhopper penguin draft genome (GCA_010082375.1 [41]) using BWA-MEM version 0.7.17 [42] with default parameters. Unmapped reads were removed using Samtools version 1.10 [43].

SNPs were identified within STACKS using *gstacks* with default parameters and only the first encountered SNP was kept in instances where multiple SNPs were identified in a single locus (--write-single-snp). Loci were filtered using VCFtools version 0.1.16 [44] to have a minimum read depth of 5, a minimum mean read depth of 15, and a minor allele count of ≥ 3. Genotypes were also filtered using PLINK 1.9 [45] where SNPs were excluded if they had more than 20% missing genotypes (--geno 0.2), and individuals were removed if they had more than 20% missing genotypes (--mind 0.2). For population genomic analyses, SNPs were further excluded if they were shown to have high levels of linkage (r^2^ ≥ 0.25) or if they were significantly out of Hardy-Weinberg equilibrium (--hwe 0.001) in at least three populations, excluding the vagrant individuals.

## ddRAD analysis

Genetic diversity summary statistics were calculated for each island population. Nucleotide diversity (π) was calculated using PLINK v.1.9 [45] using only polymorphic sites. Observed (H_O_) and expected (H_S_) heterozygosity, and the inbreeding fixation index (F_IS_) were calculated using the R package ‘hierfstat’ [46]. Weir and Cockerham’s weighted F-statistic (F_ST_) was calculated for each pair of island populations also using ‘hierfstat’ [46] and re-sampled with 1000 permutations to generate estimates of statistical significance.

Genetic structure was analysed using a Principal Component Analysis (PCA) performed using the R package ‘adegenet’ [47], a maximum likelihood estimation of individual ancestries in ADMIXTURE [48] using default parameters for number of clusters (K) from one to seven, and MCMC clustering based on co-ancestry inference in fineRADstructure [49] with a burn-in of 10,000 and 1,000,000 iterations, retaining every 100th iteration. In all instances the analysis was run on three datasets: all individuals including vagrants, individuals sampled from only the Atlantic Ocean, and individuals sampled only from the Indian Ocean.

Migration estimates were calculated between the Atlantic and Indian oceans, as well as among all island populations using BayesAss v.3.0.5 [50]. For comparison between the two oceans, all individuals were assigned to either the Atlantic Ocean or the Indian Ocean populations and allele frequency, inbreeding coefficient, and migration rate were set to 0.30, 0.50, and 0.1, respectively. For analyses between islands all individuals were assigned to their respective island population and allele frequency, inbreeding coefficient, and migration rate were set to 0.30, 0.70, and 0.1, respectively. Allele frequencies, inbreeding coefficients and migration rates were determined by parameter mixing tests where suitable values had an acceptance rate between 20% and 60%. In both instances two runs were conducted with a 1,000,000 burn-in and 10,000,000 iterations retaining every 100th iteration. Duplicate runs were checked to ensure convergence using Tracer [51].

Historical relationships between populations were investigated using TreeMix [52]. Allele frequencies were calculated using PLINK v.1.9[45] and transformed into a suitable TreeMix input file with *plink2treemix.py* (https://bitbucket.org/nygcresearch/treemix/downloads/*).* A total of 10 unrooted runs were performed for each value of migration events (M), ranging from one to seven. As all migration event values explained > 99.8% of the variance, the best fit model (m = 3) was selected based on the pairwise residual values.

## Results

### mtDNA sequencing and analysis

**Fig. 2:**
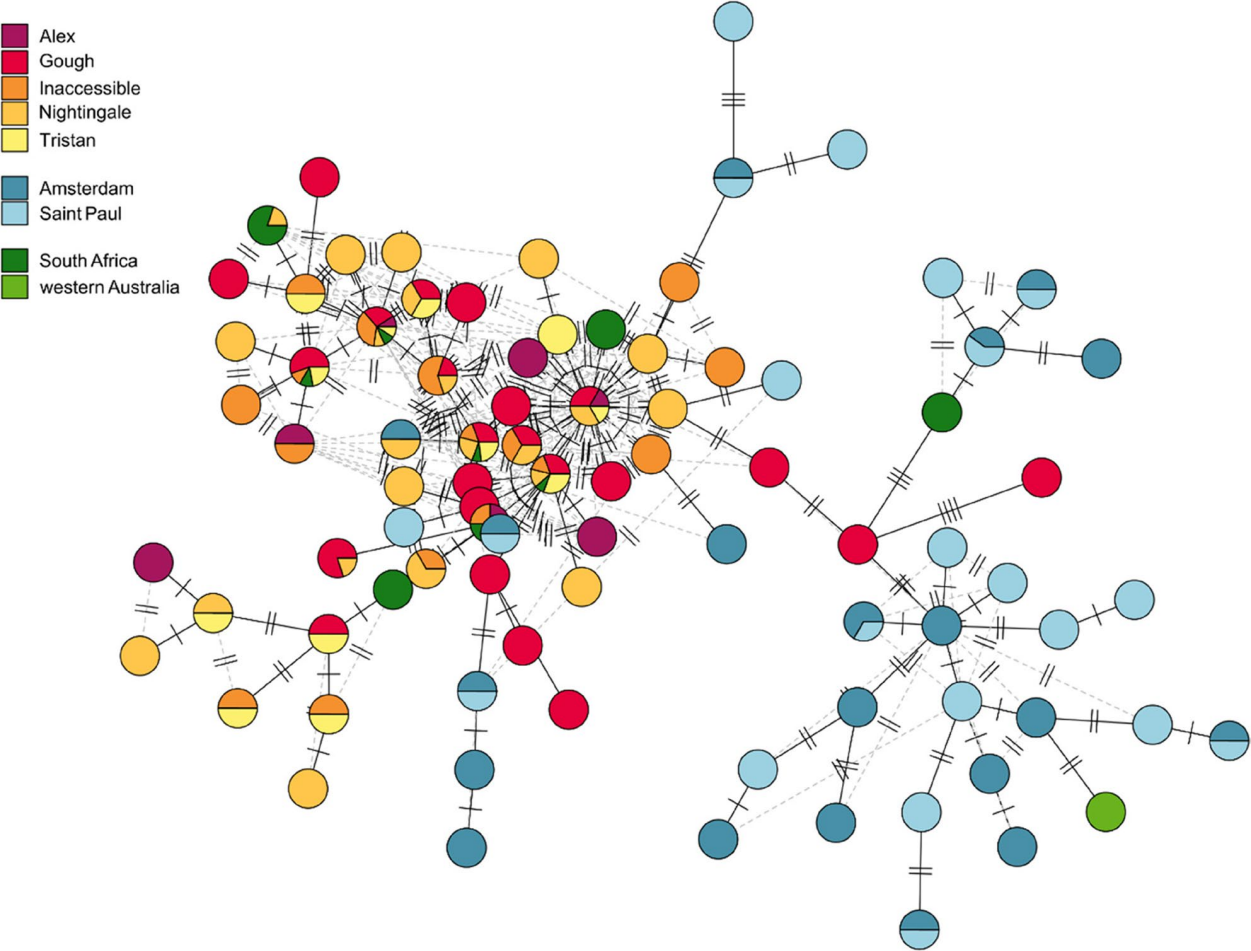
Parsimony haplotype network based on fragments of the NADH dehydrogenase 2 (ND2) gene and the control region (CR). Each of the 86 haplotypes are represented by a circle, which are not scaled by frequency of occurrence, where strikes on branches indicate the number of mutational changes between haplotypes. Haplotypes are coloured by location with Atlantic Ocean populations represented in warm colours, Indian Ocean populations represented in blues, and vagrant individuals represented in greens

Both mitochondrial loci were successfully sequenced in all 194 individuals (Supplementary Table 2). A total of 417 bp were resolved for ND2 and 299 bp were resolved for CR. A total of 15 unique ND2 haplotypes and 76 unique CR haplotypes were identified, and when combined this resulted in 86 unique haplotypes combinations. The haplotype network comprising of both ND2 and CR showed a divide between the populations in the Atlantic and the Indian oceans, although some haplotypes were shared across oceans (Fig. 2). A single branch separates the haplotypes mostly characterised in Atlantic Ocean populations and those in the Indian Ocean suggesting an initial migration event from one ocean to the other. Given the dominance of haplotypes typically seen in Atlantic Ocean populations, alongside additional satellite branches that originate with Atlantic Ocean haplotypes and terminate with haplotypes only found in the Indian Ocean, this suggests that Indian Ocean populations may have been colonised by individuals from the Atlantic Ocean. This split between oceans was more pronounced in the haplotype network comprised of just the CR locus, whereas the ND2 locus was dominated by a single haplotype with a small number of individuals possessing alternative haplotypes (Supplementary Fig. 1). In all instances, only one haplotype was shared across populations from both oceans. All vagrants sampled from South Africa clustered with the Atlantic Ocean individuals, except for one individual that clustered with birds from the Indian Ocean. The vagrant sampled from western Australia also clustered with individuals from the Indian Ocean.

## ddRAD sequencing

The number of reads sequenced per individual ranged from 9532 to 22,285,594 with an average of 6,958,427 reads. A total of six individuals did not reach the defined minimum limit of 250,000 reads and were removed from downstream analysis. The remaining 128 samples were mapped to the northern rockhopper penguin genome before a further six individuals were removed during SNP QC. An additional five individuals from South Africa were removed due to high relatedness (parent-offspring) suggesting these were captive-bred offspring. After checking genotyping rate concordance between- and within-plate control samples, only the best genotyped repeated sample was retained. This resulted in a final dataset comprising 117 individuals and 3,538 SNPs, with an average per-locus coverage ranging from 28x to 1257x.

## ddRAD analysis

Nucleotide diversity (π) ranged from 0.185 in Amsterdam and Saint Paul to 0.198 in Nightingale, with an average of 0.191 (Table 1). Values were largely consistent across all populations although values in populations in the Indian Ocean were lower than those in the Atlantic Ocean. Observed heterozygosity (H_O_) ranged from 0.143 to 0.191, with an average of 0.170. All populations exhibited H_O_ values that were lower than the expected (H_S_) values of heterozygosity, although the severity was variable across islands. Estimates of inbreeding (F_IS_) ranged from 0.039 in Amsterdam to 0.234 in Inaccessible with an average value of 0.120. In the Atlantic Ocean, low F_IS_ values were reported at Tristan da Cunha (0.051) and Alex (0.067), suggesting that there is low to no inbreeding occurring. However, F_IS_ values were markedly higher in the populations of Gough (0.219), Inaccessible (0.234), and Nightingale (0.181), suggesting that there may be some inbreeding occurring on these islands. No signature of inbreeding was detected in the Indian Ocean, where F_IS_ values were low in both the Amsterdam (0.039) and Saint Paul (0.046) populations. It is important to note that these fixation indices may be affected by overall low sequencing coverage associated with reduced representation datasets [53] although the average per-locus sequencing coverage reported here is sufficient to produce high quality genotyping.

**Table 1:** Summary statistics for each population including nucleotide diversity (π), observed heterozygosity (H_o_±SE), expected heterozygosity (H_S_±SE), and inbreeding coefficient (F_IS_). The fixation index (F_ST_) between pairs of populations is also shown where an asterisk indicates significance at *p* ≤ 0.05

					Atlantic					Indian	
					Alex	Gough	Inaccessible	Nightingale	Tristan	Amsterdam	Saint Paul
	π	H_o_	H_S_	F_IS_	F_ST_						
Alex	0.187 ± 0.003	0.184 ± 0.003	0.204 ± 0.003	0.067 ± 0.007	-	0.000*	0.000	−0.003	0.001	0.048*	0.049*
Gough	0.194 ± 0.003	0.150 ± 0.003	0.204 ± 0.003	0.219 ± 0.007		-	0.007*	0.004*	0.005	0.049*	0.051*
Inaccessible	0.191 ± 0.003	0.143 ± 0.003	0.203 ± 0.003	0.234 ± 0.007			-	0.003	0.003	0.051*	0.051*
Nightingale	0.198 ± 0.003	0.161 ± 0.003	0.205 ± 0.003	0.181 ± 0.006				-	0.000	0.047*	0.048*
Tristan	0.197 ± 0.003	0.191 ± 0.003	0.204 ± 0.003	0.051 ± 0.005					-	0.047*	0.049*
Amsterdam	0.185 ± 0.003	0.181 ± 0.003	0.191 ± 0.003	0.039 ± 0.005						-	0.000
Saint Paul	0.185 ± 0.003	0.180 ± 0.003	0.191 ± 0.003	0.046 ± 0.005							-

**Fig. 3:**
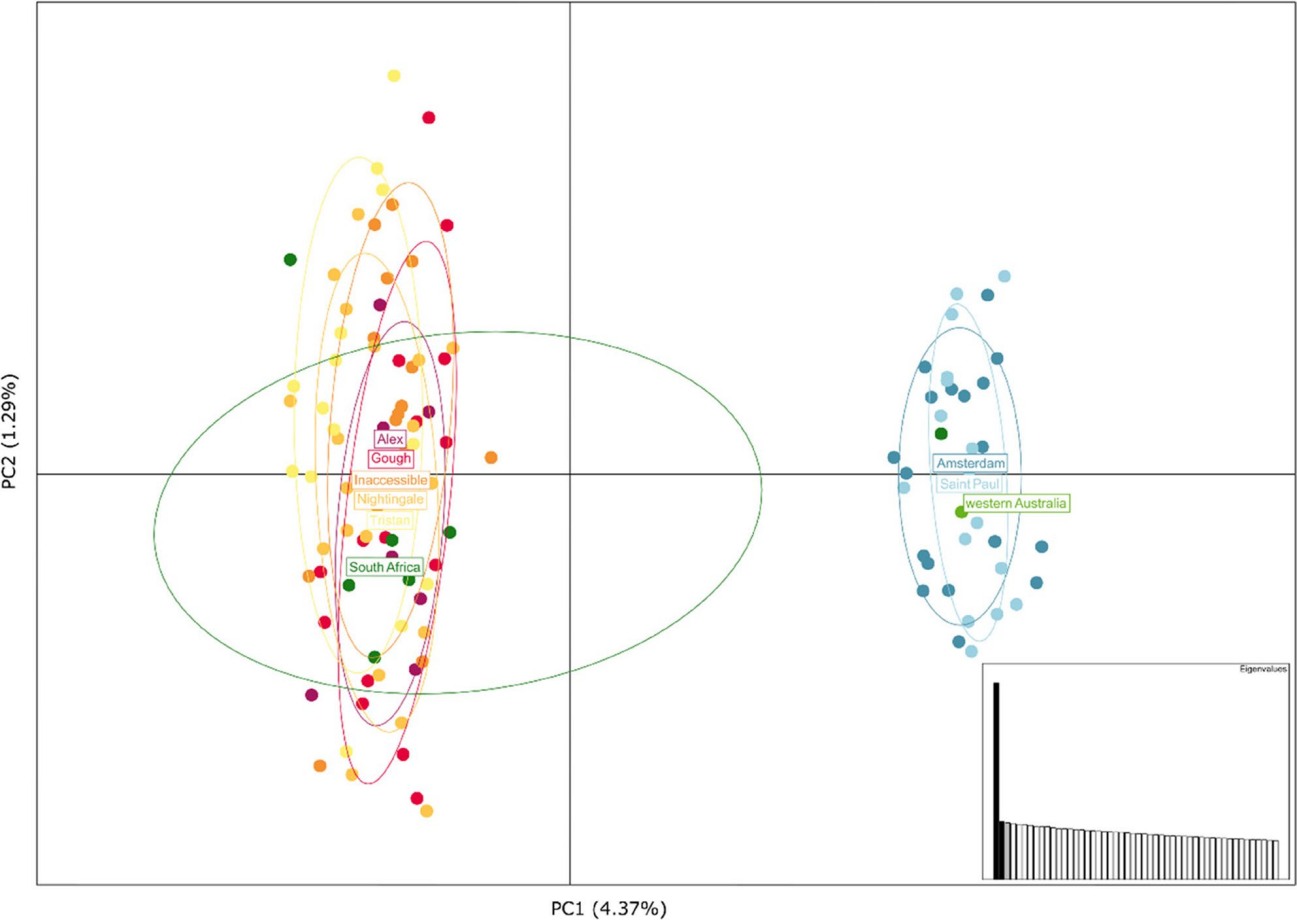
Principal components analysis of 117 northern rockhopper penguins sampled from five islands in the Atlantic Ocean, two islands in the Indian Ocean, and vagrant individuals from South Africa and western Australia. Individuals are coloured by location with Atlantic Ocean populations represented in warm colours, Indian Ocean populations represented in blues, and vagrant individuals represented in greens. Eigenvalues showing the proportion of variance explained by each principal component are presented in the insert

**Fig. 4:**
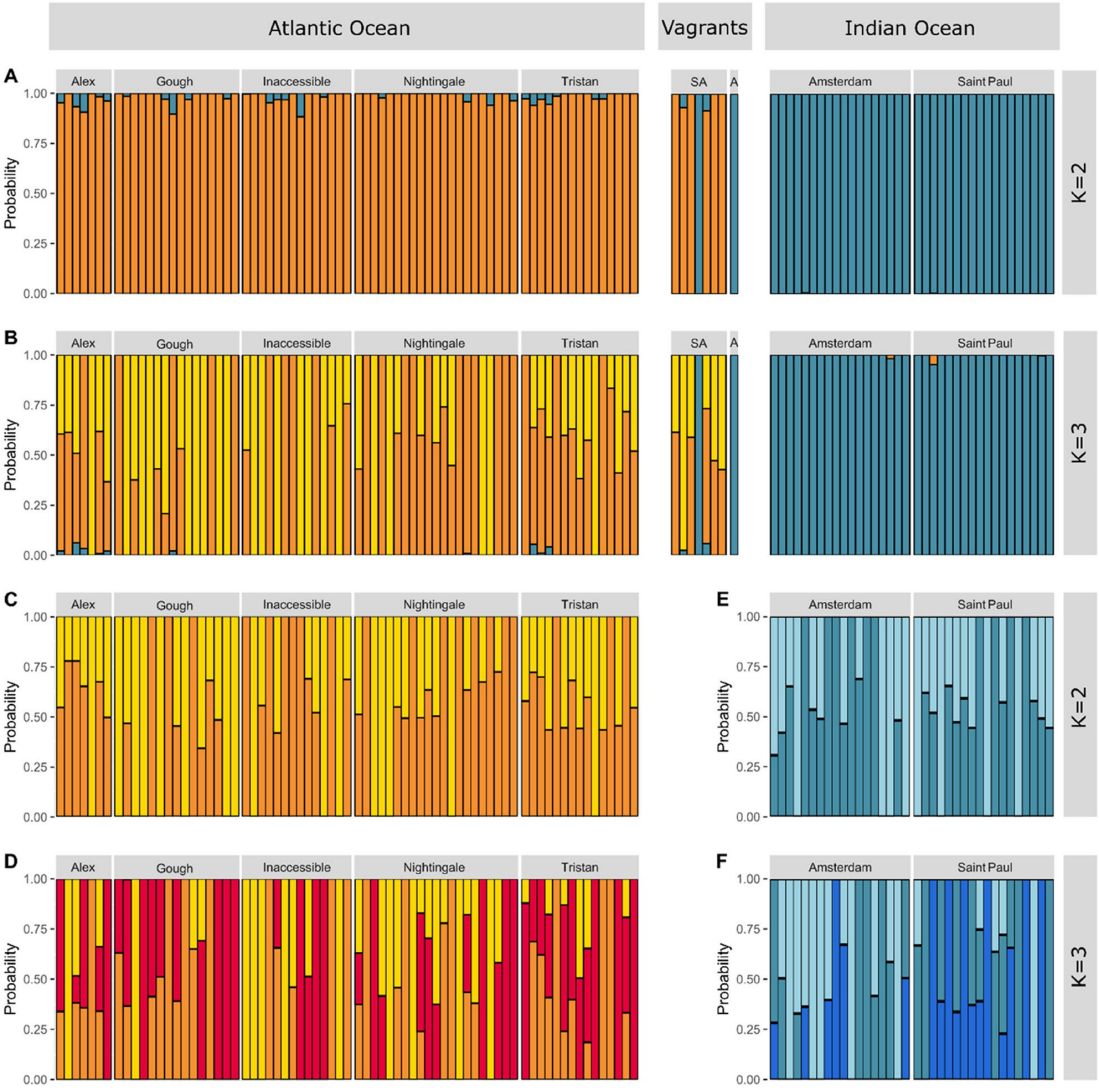
ADMIXTURE plots for all colonies and vagrant individuals at two (**A**) and three (**B**) clustering groups, only Atlantic Ocean colonies at two (**C**) and three (**D**) clustering groups, and only Indian Ocean colonies at two (**E**) and three (**F**) clustering groups. Each vertical bar represents one individual where the proportion of colour indicates inferred membership proportions to genetic clusters. The vagrants denoted as SA are from South Africa, and the vagrant individual denoted as A is from western Australia

All methods used to analyse population structure indicated differentiation between the penguins in the Atlantic and Indian oceans. Pairwise F_ST_ values calculated between all island populations showed population differentiation ranging from − 0.003 to 0.051 with the only significant values of pairwise F_ST_ seen between populations in the Atlantic and the Indian oceans (F_ST_ = 0.046, *p* ≤ 0.05; Table 1). In the Principal Component Analysis (PCA) the first axis split the samples between the penguins in the two oceans (Fig. 3) and the ADMIXTURE analysis also clustered the samples into Atlantic Ocean and Indian Ocean clades at k = 2 (Fig. 4). No further substructure was observed in any of the analyses, suggesting geneflow between the islands within each ocean (Fig. 4; Supplementary Fig. 2).

FineRADstructure corroborated the lack of further structure, showing that individuals only clustered by ocean (Supplementary Fig. 3). The vagrant individual from western Australia clustered with the Indian Ocean populations in all analyses, while all but one vagrant individual sampled from South Africa clustered with the Atlantic Ocean populations (Figs. 3 and 4A).

**Fig. 5:**
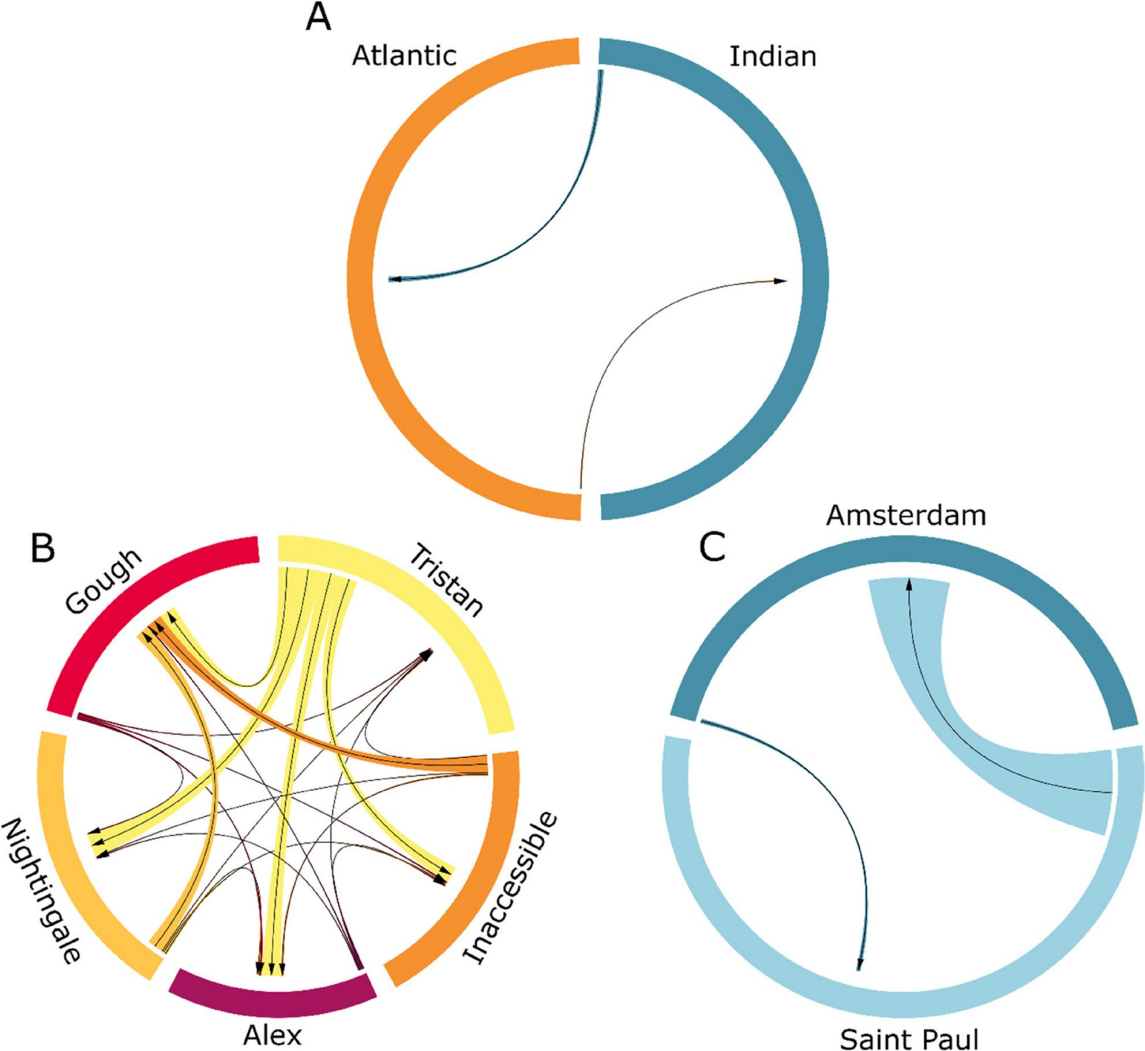
Migration estimates between (**A**) the two identified genetic clusters in the Atlantic and Indian oceans, (**B**) all islands in the Atlantic Ocean, and (**C**) all islands in the Indian Ocean. Arrows show the direction of migration and ribbons are scaled by the relative amount of migration estimated

Migration estimates within and between the islands in the Atlantic and Indian Ocean were low overall (Fig. 5; Supplementary Table 3). Migration between the two identified genetic clusters of the Atlantic and Indian oceans was low in both directions with slightly higher migration from the Indian to the Atlantic Ocean (0.0178) than from the Atlantic to the Indian Ocean (0.0045) although the 95% confidence intervals included zero so it may also be possible that there is no gene flow between the oceans. Migration estimates between islands in the Atlantic Ocean ranged from 0.0118 (Gough to Nightingale) to 0.2228 (Tristan to Nightingale). Within the Atlantic Ocean, the largest number of migrants to other populations were from Tristan, and there was little immigration, although this may be an artifact of very low F_ST_ (< 0.05) producing unreliable migration estimates [54]. In contrast, Gough received migrants from all the other Atlantic islands with little emigration. In the Indian Ocean, considerably more emigration occurred from Saint Paul to Amsterdam (0.2539) than from Amsterdam to Saint Paul (0.0132).

**Fig. 6:**
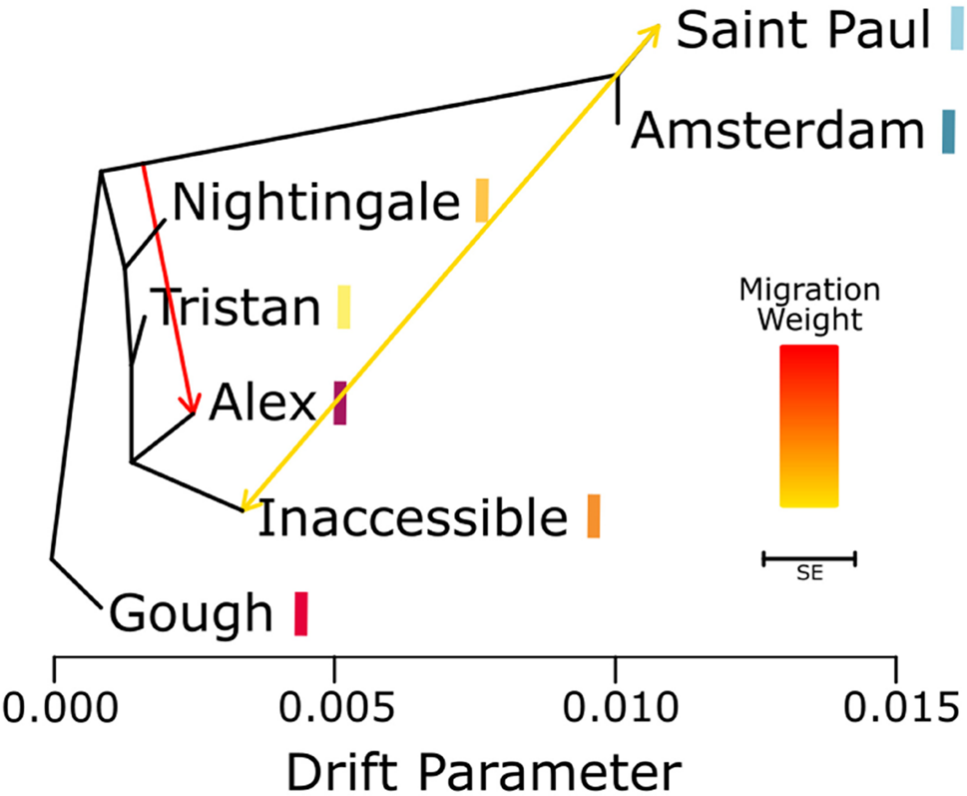
Bifurcating maximum likelihood tree produced by TreeMix with three migration events. The migration weight scale depicts the relative amount of migration with arrows indicating direction of migration. The SE scale bar represents 10 times the average standard error within the sample covariance matrix. Populations are coloured by location with Atlantic Ocean populations represented in warm colours and Indian Ocean populations represented in blues

The TreeMix phylogeny grouped the Atlantic Ocean islands except for Gough, the most distant population, and grouped the Indian Ocean islands together (Fig. 6). A large migration event from an ancestral population to the Atlantic Ocean, specifically to Alex Island, was identified; however, this may be an artefact of the small number of samples from that island. A smaller degree of reciprocal migration was also found between the two oceans, particularly between Inaccessible Island in the Atlantic Ocean and Saint Paul Island in the Indian Ocean.

## Discussion

 This study provides the first comprehensive genetic population structure analysis of northern rockhopper penguins across all seven islands inhabited with breeding birds, and provides vital knowledge for conservation management of the species. Here we show using molecular data that island populations in the Atlantic Ocean are genetically differentiated from those in the Indian Ocean, and that there is very little migration between the ocean basins. However, vagrant individuals found in Cape Town, South Africa have been shown to originate from both the Atlantic and the Indian Ocean populations suggesting there is potential for occasional mixing. No distinct substructure was shown between island populations within each ocean basin, although the extent of gene flow between these islands is unclear. These findings emphasis the need to fully understand connectivity between island populations for determining appropriate management units for effective conservation management strategies.

Several lines of investigation suggest the northern rockhopper penguin is split into two genetically distinct clusters grouped by ocean. Mitochondrial haplotype data showed an overall split between oceans with some shared haplotypes where the control region alone showed a rosette shape formed from individuals in the Atlantic Ocean with a single branch extending to haplotypes only found in individuals from the Indian Ocean. This suggests that the geologically younger islands of Amsterdam and Saint Paul in the Indian Ocean (0.4 and < 0.2 MYA, respectively) were colonised by individuals from the Atlantic Ocean which has previously been hypothesised as a colonisation route due to prevailing easterly surface currents [55]. Nuclear data showed very clear segregation based on ocean provenance which is likely maintained due to the relatively small intra-ocean geographical distances between breeding islands (c. 0.25 to 400 km in the Atlantic Ocean, and c. 100 km in the Indian Ocean) compared to the large inter-ocean geographical distance (> 5000 km). A limited amount of contemporary admixture was detected alongside low levels of gene flow between island groups suggesting that long-distance sporadic dispersal may still occur, although at a low level.

Notwithstanding, vagrant individuals were found to have originated from colonies in both the Atlantic and Indian oceans. South Africa is located between the two ocean island groups and vagrants were found to have originated from both ocean basins, with the majority originating from the Atlantic Ocean, as might be expected from the much larger Atlantic population [1] and prevailing easterly ocean current systems [55]. The vagrant penguin from western Australia originated from one of the islands in the Indian Ocean which is intuitive given their geographical proximity and that penguins from Amsterdam have been shown to forage longitudinally as far as the south-western coast of Australia [56]. Long ranging migration events have been previously documented where a vagrant northern rockhopper penguin found on the Kerguelen Archipelago (southern Indian Ocean) was genetically identified as having originated from the Atlantic Ocean island group situated over 6000 km away [5]. In addition, penguins from Amsterdam Island have been tracked as far as 2200 km away from the colony post-moult [57] suggesting they are capable of traversing large geographical distances. Nevertheless, long-distance migrations are likely to be infrequent as tracking of rockhopper penguins from Tristan and Gough indicates that the birds remain predominantly within the South Atlantic [57, 58]. No evidence of within-ocean substructure was detected but estimates of migration between islands were relatively low and uneven between populations. Within both ocean basins migration estimates between islands were low overall but even one migrant per generation should be sufficient to maintain gene flow [59]. The level and direction of migration varied between islands with the most notable signature being considerably more migrants shown to move from Saint Paul to Amsterdam than vice-versa. While this signature contradicts a previous pattern showing penguins migrating in a south easterly direction from Amsterdam after breeding [56] it may, in part, be due to population expansion in the smaller island of Saint Paul (8 km^2^) which increased 95.6% between 1971 and 2018[9, 10] coupled with the population decline on the larger island of Amsterdam (58 km^2^) where penguin numbers decreased ~ 80% between 1972 and 2015(9) with a further decrease of 66% between 2015 and 2022 [11]. However, it is important to note that our migration estimates exhibited large 95% confidence intervals due to the low levels of differentiation between island populations.

These patterns of population structure are similar to those reported for some populations of northern rockhopper penguins and for many other penguin species. The low but significant levels of differentiation between ocean basin populations of northern rockhopper penguins calculated here are in line with F_ST_ values previously reported between the islands of Amsterdam in the Indian Ocean and Gough [30] and Nightingale [31] in the Atlantic Ocean, and the lack of differentiation between island populations in the same ocean basin was also previously reported between Gough and Nightingale in the Atlantic Ocean [33]. While strong signatures of population structuring have been observed in Humboldt (*Spheniscus humboldti*) [60] and gentoo penguins (*Pygoscelis papua*) [61], other penguin species including emperor (*Aptenodytes forsteri*) [61, 62], king (*A. patagonicus*) [61], chinstrap (*P. antarcticus*) [61], Adélie (*P. adeliae*) [61] and southern rockhopper (*E. chrysocome*) [32, 63] exhibit low but significant differentiation between populations comparable to our results. Notably, southern rockhopper penguins were shown to differentiate into two major clades with no evidence of any further fine-scale structuring [63] similar to the patterns of differentiation seen here in the northern rockhopper penguins.

Genetic diversity and estimates of inbreeding varied among islands and between oceans. Estimates of genetic diversity were in agreement with previous values calculated for northern rockhopper penguins on the islands of Nightingale and Amsterdam [32] which showed their genetic diversity to be lower than values seen in the southern rockhopper and the eastern rockhopper (*E. filholi*) [32]. Genetic diversity was estimated to be lower for populations in the Indian Ocean compared to those in the Atlantic, which may be due to smaller population sizes in the Indian Ocean islands [2]. The overall trend of low genetic diversity may be a signature of loss of genetic diversity due to extensive population declines and genetic bottlenecks with the number of birds breeding on Gough Island estimated to have declined by 98% between 1955 and 2006(2, 9, 10) and global trends show an overall decline of > 50% decline in the Atlantic population between 1975 and 2005(2). This might also explain the evidence of inbreeding seen in several of these populations with Gough, Inaccessible and Nightingale showing the most pronounced signatures of inbreeding.

## Conclusions

Our results indicate that the northern rockhopper penguin populations breeding in each ocean basin should be managed as separate management units reflecting their clear genetic differentiation and geographical separation. By recognising them as distinct management units this provides a clear framework for assessing and mitigating risks specific to each ocean basin. As an example, the potential disease risk from *Pasteurella multocida* and *Erysipelothrix amsterdamsensis* on Amsterdam island [18, 64] is likely to only affect islands within the Indian Ocean. Nevertheless, continued monitoring and risk assessment is necessary as the populations on each island face a different suite of demographic and environmental pressures; differential signatures of inbreeding suggest some colonies are at higher risk of reduced genetic fitness where maintaining genetically healthy populations across all islands is imperative to the persistence of these colonies. Continued monitoring of demographic trends within management units and across all islands will be essential to identify emerging risks and guide species management decisions.

## Supplementary Information

Supplementary Material 1.

## Data Availability

All data used in this study are available via NCBI GenBank and the Tristan Data portal. Mitochondrial data are found in accession numbers PX216465-PX216555 and Supplementary Table 2. Raw ddRAD sequences are found in BioProject: PRJNA1309037 (BioSamples: SAMN50726567– SAMN50726683).

## Abbreviations

BWA: Burrow-Wheeler Aligner
CEFAS: Centre for Environment, Fisheries and Aquaculture Science
CR: Control Region
ddRAD: double-digest Restriction-site Associated DNA
DNA: Deoxyribonucleic Acid
EDTA: Ethylenediaminetetraacetic acid
FTA: Fast Technology for Analysis
IUCN: International Union for Conservation of Nature
MAFFT: Multiple Alignment using Fast Fourier Transform
mtDNA: mitochondrial DNA
MYA: Millions of Years Ago
ND2: NADH dehydrogenase 2
PCA: Principal Component Analysis
PCR: Polymerase Chain Reaction
RNN: Réserve Naturelle Nationale
RSPB: Royal Society for the Protection of Birds
RZSS: Royal Zoological Society of Scotland
SNP: Single Nucleotide Polymorphism
TAAF: Terres Australes et Antarctiques Françaises
