# Supplementary material for: Uncovering genetic population structure in the Endangered northern rockhopper penguin (Eudyptes moseleyi) across islands in the Southern Atlantic and Indian oceans
Source: BMC Genomics. 2026 Jan 6;27:134. doi: 10.1186/s12864-025-12487-9 (PMC12870283; doi:10.1186/s12864-025-12487-9)
Supplement: Supplementary file 1 — Supplementary Material 1. [file 12864_2025_12487_MOESM1_ESM.docx]

**
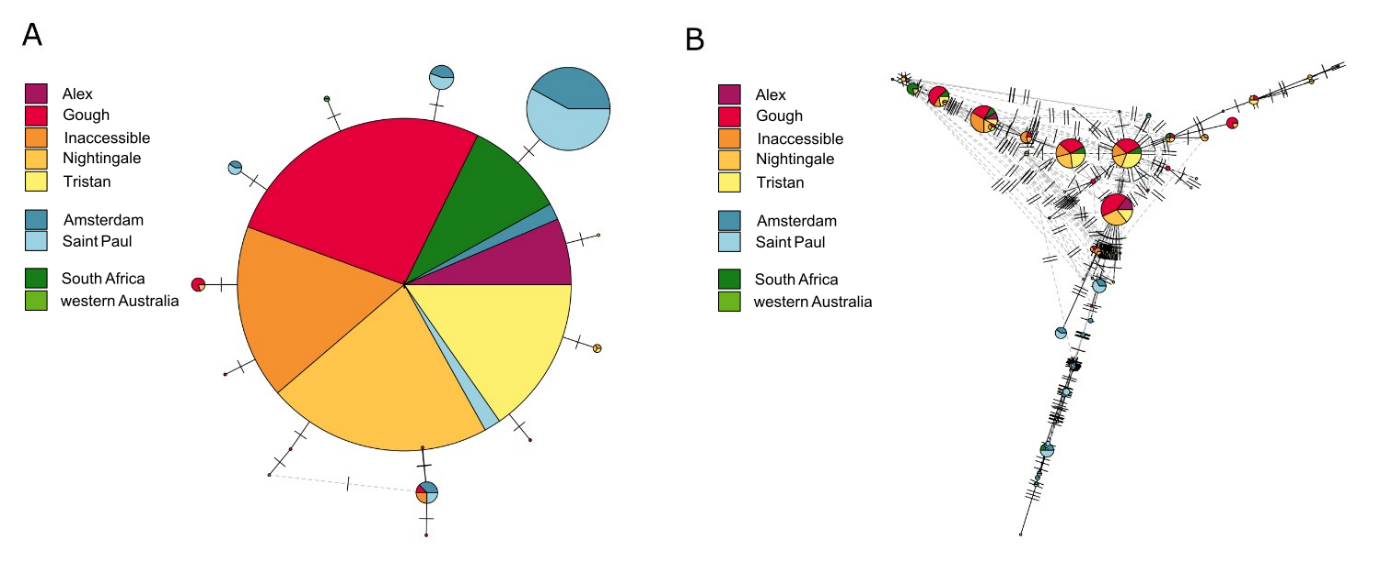
Supplementary Figure 1.** Parsimony haplotype networks based on fragments of the A) NADH dehydrogenase 2 (ND2) gene and B) the control region (CR). Each of the haplotypes are represented by a circle, which are scaled by frequency of occurrence, where strikes on branches indicate the number of mutational changes between haplotypes. Haplotypes are coloured by location with Atlantic Ocean populations represented in warm colours, Indian Ocean populations represented in blues, and vagrant individuals represented in greens.

**
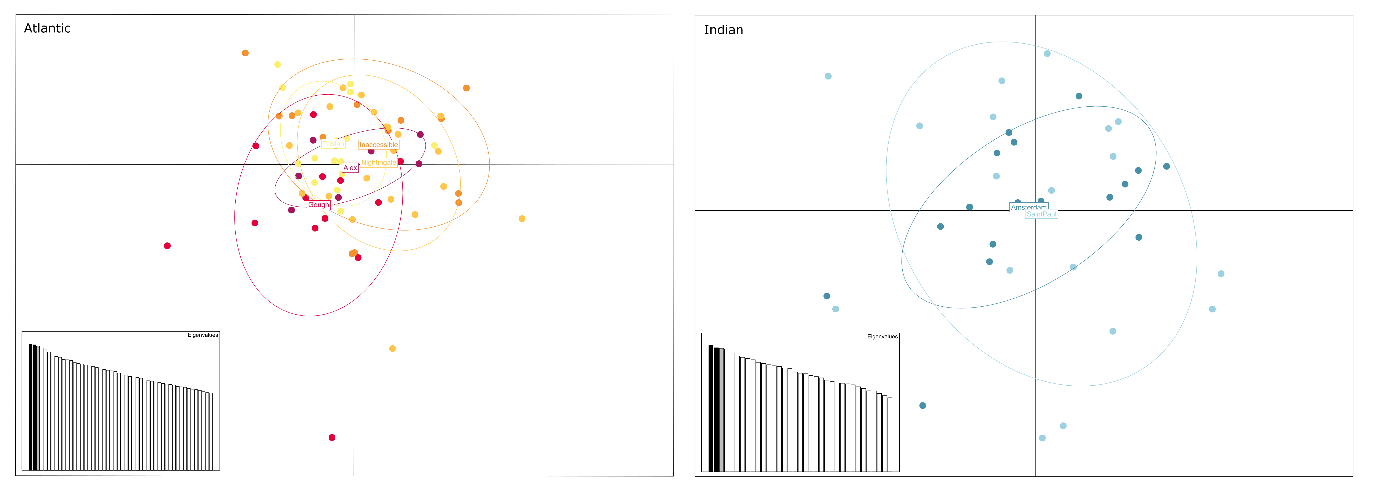
**

**Supplementary Figure 2.** Principal components analysis of Northern rockhopper penguin populations from five islands in the Atlantic Ocean and two islands in the Indian Ocean. Individuals are coloured by location with Atlantic Ocean populations represented in warm colours and Indian Ocean populations represented in blues.

**
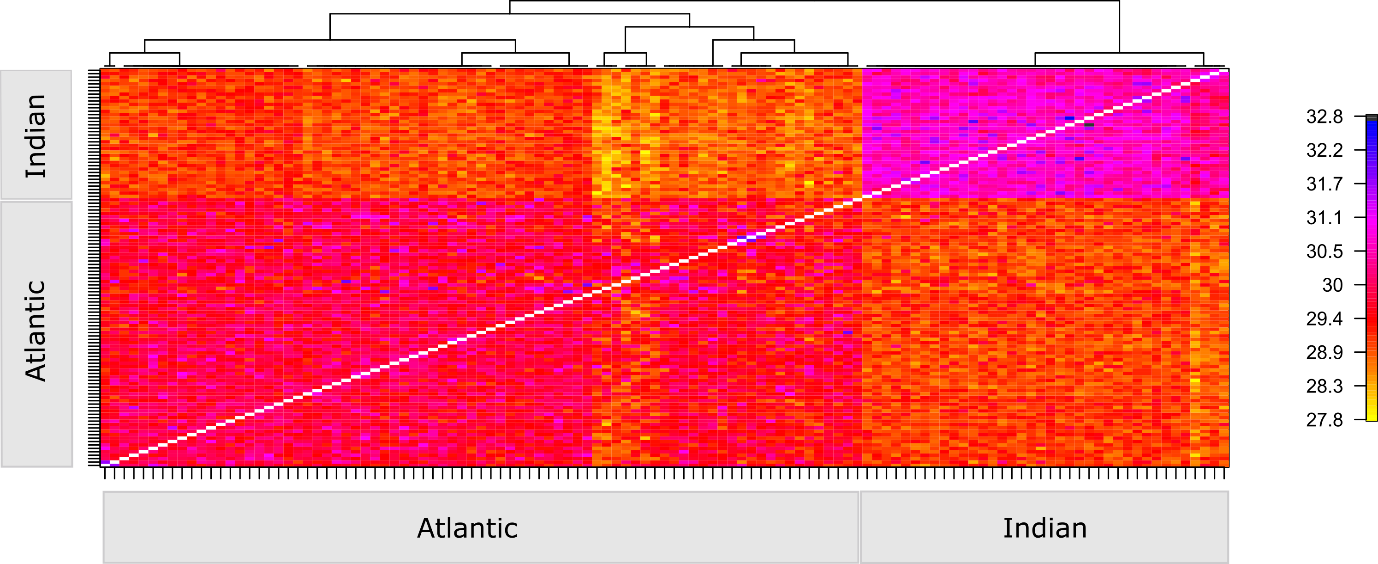
**

**Supplementary Figure 3.** Clustered fineRADstructure matrix of co-ancestry coefficients. Each square represents the co-ancestry coefficient generated between two individuals which is scaled from less to more shared co-ancestry.

**Supplementary Table 1.** Sample information for Northern rockhopper individuals used in this study with the number of samples successfully sequenced at two mitochondrial loci, the number of samples sequenced using ddRAD, and the number of ddRAD samples that passed QC and were used for downstream analysis.

|  | **Population** | **mtDNA** | **ddRAD sequenced** | **ddRAD passed QC** |
| --- | --- | --- | --- | --- |
| **Atlantic Ocean** | **Alex** | 8 | 7 | 7 |
|  | **Gough** | 43 | 20 | 16 |
|  | **Inaccessible** | 24 | 20 | 14 |
|  | **Nightingale** | 30 | 23 | 21 |
|  | **Tristan da Cunha** | 20 | 15 | 15 |
| **Indian Ocean** | **Amsterdam** | 26 | 18 | 18 |
|  | **Saint Paul** | 30 | 18 | 18 |
| **Vagrants** | **western Australia** | 1 | 1 | 1 |
|  | **South Africa** | 12 | 12 | 7 |

**Supplementary Table 2.** Haplotype data for all sequenced individuals including individual ID and location data.

|  |  | **NADH dehydrogenase 2 (ND2)** | | **Control region (CR)** | |
| --- | --- | --- | --- | --- | --- |
| **Population** | **Individual ID** | **Haplotype** | **NCBI Ref** | **Haplotype** | **NCBI Ref** |
| Alex | PEN403 | A | PX216465 | BL | PX216543 |
| Alex | PEN404 | A | PX216465 | BV | PX216553 |
| Alex | PEN405 | A | PX216465 | BW | PX216554 |
| Alex | PEN406 | A | PX216465 | B | PX216481 |
| Alex | PEN407 | A | PX216465 | AM | PX216518 |
| Alex | PEN408 | A | PX216465 | BX | PX216555 |
| Alex | PEN409 | A | PX216465 | B | PX216481 |
| Alex | PEN410 | A | PX216465 | A | PX216480 |
| Gough | PEN036 | A | PX216465 | E | PX216484 |
| Gough | PEN037 | A | PX216465 | E | PX216484 |
| Gough | PEN038 | A | PX216465 | C | PX216482 |
| Gough | PEN039 | J | PX216474 | H | PX216487 |
| Gough | PEN040 | A | PX216465 | N | PX216493 |
| Gough | PEN041 | A | PX216465 | E | PX216484 |
| Gough | PEN042 | L | PX216476 | O | PX216494 |
| Gough | PEN043 | A | PX216465 | AL | PX216517 |
| Gough | PEN044 | E | PX216469 | AX | PX216529 |
| Gough | PEN045 | O | PX216479 | B | PX216481 |
| Gough | PEN046 | A | PX216465 | BP | PX216547 |
| Gough | PEN047 | M | PX216477 | BD | PX216535 |
| Gough | PEN048 | A | PX216465 | N | PX216493 |
| Gough | PEN049 | A | PX216465 | BK | PX216542 |
| Gough | PEN050 | A | PX216465 | B | PX216481 |
| Gough | PEN051 | A | PX216465 | V | PX216501 |
| Gough | PEN052 | A | PX216465 | C | PX216482 |
| Gough | PEN053 | A | PX216465 | G | PX216486 |
| Gough | PEN054 | A | PX216465 | Z | PX216505 |
| Gough | PEN055 | J | PX216474 | H | PX216487 |
| Gough | PEN056 | A | PX216465 | D | PX216483 |
| Gough | PEN057 | A | PX216465 | C | PX216482 |
| Gough | PEN058 | K | PX216475 | O | PX216494 |
| Gough | PEN059 | J | PX216474 | H | PX216487 |
| Gough | PEN060 | A | PX216465 | D | PX216483 |
| Gough | PEN061 | A | PX216465 | P | PX216495 |
| Gough | PEN062 | A | PX216465 | P | PX216495 |
| Gough | PEN063 | A | PX216465 | A | PX216480 |
| Gough | PEN064 | A | PX216465 | BQ | PX216548 |
| Gough | PEN065 | D | PX216468 | B | PX216481 |
| Gough | PEN066 | A | PX216465 | E | PX216484 |
| Gough | PEN067 | A | PX216465 | AB | PX216507 |
| Gough | PEN068 | J | PX216474 | H | PX216487 |
| Gough | PEN069 | A | PX216465 | C | PX216482 |
| Gough | PEN070 | A | PX216465 | A | PX216480 |
| Gough | PEN071 | A | PX216465 | D | PX216483 |
| Gough | PEN072 | A | PX216465 | D | PX216483 |
| Gough | PEN073 | A | PX216465 | E | PX216484 |
| Gough | PEN074 | A | PX216465 | A | PX216480 |
| Gough | PEN075 | A | PX216465 | B | PX216481 |
| Gough | PEN076 | A | PX216465 | B | PX216481 |
| Gough | PEN077 | A | PX216465 | B | PX216481 |
| Gough | PEN078 | A | PX216465 | AI | PX216514 |
| Inaccessible | PEN107 | A | PX216465 | E | PX216484 |
| Inaccessible | PEN108 | A | PX216465 | G | PX216486 |
| Inaccessible | PEN109 | A | PX216465 | C | PX216482 |
| Inaccessible | PEN110 | A | PX216465 | A | PX216480 |
| Inaccessible | PEN111 | C | PX216467 | AB | PX216507 |
| Inaccessible | PEN112 | A | PX216465 | G | PX216486 |
| Inaccessible | PEN113 | A | PX216465 | A | PX216480 |
| Inaccessible | PEN114 | A | PX216465 | M | PX216492 |
| Inaccessible | PEN115 | A | PX216465 | BH | PX216539 |
| Inaccessible | PEN116 | A | PX216465 | BL | PX216543 |
| Inaccessible | PEN117 | A | PX216465 | AM | PX216518 |
| Inaccessible | PEN118 | A | PX216465 | A | PX216480 |
| Inaccessible | PEN119 | A | PX216465 | BM | PX216544 |
| Inaccessible | PEN120 | A | PX216465 | C | PX216482 |
| Inaccessible | PEN121 | K | PX216475 | Y | PX216504 |
| Inaccessible | PEN122 | A | PX216465 | Z | PX216505 |
| Inaccessible | PEN123 | A | PX216465 | BJ | PX216541 |
| Inaccessible | PEN124 | A | PX216465 | AG | PX216512 |
| Inaccessible | PEN125 | A | PX216465 | AF | PX216511 |
| Inaccessible | PEN126 | K | PX216475 | Y | PX216504 |
| Inaccessible | PEN127 | A | PX216465 | D | PX216483 |
| Inaccessible | PEN128 | A | PX216465 | G | PX216486 |
| Inaccessible | PEN129 | A | PX216465 | D | PX216483 |
| Inaccessible | PEN132 | A | PX216465 | A | PX216480 |
| Nightingale | PEN079 | A | PX216465 | M | PX216492 |
| Nightingale | PEN080 | A | PX216465 | A | PX216480 |
| Nightingale | PEN081 | B | PX216466 | A | PX216480 |
| Nightingale | PEN082 | A | PX216465 | B | PX216481 |
| Nightingale | PEN083 | A | PX216465 | AE | PX216510 |
| Nightingale | PEN084 | A | PX216465 | BR | PX216549 |
| Nightingale | PEN085 | A | PX216465 | BC | PX216534 |
| Nightingale | PEN086 | A | PX216465 | BI | PX216540 |
| Nightingale | PEN087 | A | PX216465 | AI | PX216514 |
| Nightingale | PEN088 | A | PX216465 | X | PX216503 |
| Nightingale | PEN089 | C | PX216467 | AD | PX216509 |
| Nightingale | PEN090 | A | PX216465 | B | PX216481 |
| Nightingale | PEN091 | A | PX216465 | AJ | PX216515 |
| Nightingale | PEN092 | A | PX216465 | BG | PX216538 |
| Nightingale | PEN093 | A | PX216465 | X | PX216503 |
| Nightingale | PEN094 | A | PX216465 | D | PX216483 |
| Nightingale | PEN095 | A | PX216465 | D | PX216483 |
| Nightingale | PEN096 | A | PX216465 | M | PX216492 |
| Nightingale | PEN097 | A | PX216465 | G | PX216486 |
| Nightingale | PEN098 | A | PX216465 | C | PX216482 |
| Nightingale | PEN099 | A | PX216465 | C | PX216482 |
| Nightingale | PEN100 | A | PX216465 | AH | PX216513 |
| Nightingale | PEN101 | A | PX216465 | BS | PX216550 |
| Nightingale | PEN102 | J | PX216474 | H | PX216487 |
| Nightingale | PEN103 | A | PX216465 | B | PX216481 |
| Nightingale | PEN104 | A | PX216465 | T | PX216499 |
| Nightingale | PEN105 | A | PX216465 | C | PX216482 |
| Nightingale | PEN106 | A | PX216465 | BT | PX216551 |
| Nightingale | PEN130 | A | PX216465 | B | PX216481 |
| Nightingale | PEN131 | A | PX216465 | Z | PX216505 |
| Tristan | PEN383 | A | PX216465 | B | PX216481 |
| Tristan | PEN384 | A | PX216465 | C | PX216482 |
| Tristan | PEN385 | A | PX216465 | BU | PX216552 |
| Tristan | PEN386 | A | PX216465 | D | PX216483 |
| Tristan | PEN387 | A | PX216465 | E | PX216484 |
| Tristan | PEN388 | A | PX216465 | AB | PX216507 |
| Tristan | PEN389 | A | PX216465 | D | PX216483 |
| Tristan | PEN390 | A | PX216465 | B | PX216481 |
| Tristan | PEN391 | A | PX216465 | AF | PX216511 |
| Tristan | PEN392 | A | PX216465 | A | PX216480 |
| Tristan | PEN393 | A | PX216465 | D | PX216483 |
| Tristan | PEN394 | A | PX216465 | E | PX216484 |
| Tristan | PEN395 | A | PX216465 | BL | PX216543 |
| Tristan | PEN396 | A | PX216465 | C | PX216482 |
| Tristan | PEN397 | A | PX216465 | AI | PX216514 |
| Tristan | PEN398 | A | PX216465 | D | PX216483 |
| Tristan | PEN399 | A | PX216465 | AE | PX216510 |
| Tristan | PEN400 | A | PX216465 | AG | PX216512 |
| Tristan | PEN401 | A | PX216465 | C | PX216482 |
| Tristan | PEN402 | C | PX216467 | AB | PX216507 |
| Amsterdam | PEN140 | H | PX216472 | AO | PX216520 |
| Amsterdam | PEN141 | H | PX216472 | AZ | PX216531 |
| Amsterdam | PEN142 | F | PX216470 | AS | PX216524 |
| Amsterdam | PEN143 | H | PX216472 | BA | PX216532 |
| Amsterdam | PEN144 | H | PX216472 | AA | PX216506 |
| Amsterdam | PEN145 | H | PX216472 | AW | PX216528 |
| Amsterdam | PEN146 | K | PX216475 | I | PX216488 |
| Amsterdam | PEN147 | H | PX216472 | AN | PX216519 |
| Amsterdam | PEN148 | A | PX216465 | U | PX216500 |
| Amsterdam | PEN149 | K | PX216475 | Q | PX216496 |
| Amsterdam | PEN150 | F | PX216470 | F | PX216485 |
| Amsterdam | PEN151 | G | PX216471 | BE | PX216536 |
| Amsterdam | PEN152 | H | PX216472 | AA | PX216506 |
| Amsterdam | PEN154 | H | PX216472 | J | PX216489 |
| Amsterdam | PEN155 | H | PX216472 | S | PX216498 |
| Amsterdam | PEN156 | H | PX216472 | S | PX216498 |
| Amsterdam | PEN159 | N | PX216478 | Q | PX216496 |
| Amsterdam | PEN160 | F | PX216470 | BB | PX216533 |
| Amsterdam | PEN161 | F | PX216470 | F | PX216485 |
| Amsterdam | PEN162 | A | PX216465 | T | PX216499 |
| Amsterdam | PEN164 | I | PX216473 | L | PX216491 |
| Amsterdam | PEN165 | H | PX216472 | AU | PX216526 |
| Amsterdam | PEN166 | H | PX216472 | J | PX216489 |
| Amsterdam | PEN167 | H | PX216472 | W | PX216502 |
| Amsterdam | PEN168 | K | PX216475 | I | PX216488 |
| Amsterdam | PEN199 | I | PX216473 | L | PX216491 |
| Saint Paul | PEN169 | F | PX216470 | F | PX216485 |
| Saint Paul | PEN170 | F | PX216470 | BB | PX216533 |
| Saint Paul | PEN171 | H | PX216472 | J | PX216489 |
| Saint Paul | PEN172 | H | PX216472 | R | PX216497 |
| Saint Paul | PEN173 | K | PX216475 | I | PX216488 |
| Saint Paul | PEN174 | F | PX216470 | F | PX216485 |
| Saint Paul | PEN175 | H | PX216472 | AT | PX216525 |
| Saint Paul | PEN176 | I | PX216473 | L | PX216491 |
| Saint Paul | PEN177 | A | PX216465 | U | PX216500 |
| Saint Paul | PEN178 | H | PX216472 | R | PX216497 |
| Saint Paul | PEN179 | H | PX216472 | AP | PX216521 |
| Saint Paul | PEN180 | H | PX216472 | AW | PX216528 |
| Saint Paul | PEN181 | H | PX216472 | K | PX216490 |
| Saint Paul | PEN182 | I | PX216473 | AC | PX216508 |
| Saint Paul | PEN183 | H | PX216472 | J | PX216489 |
| Saint Paul | PEN184 | H | PX216472 | K | PX216490 |
| Saint Paul | PEN185 | F | PX216470 | F | PX216485 |
| Saint Paul | PEN186 | H | PX216472 | K | PX216490 |
| Saint Paul | PEN187 | H | PX216472 | AY | PX216530 |
| Saint Paul | PEN188 | H | PX216472 | AR | PX216523 |
| Saint Paul | PEN189 | H | PX216472 | R | PX216497 |
| Saint Paul | PEN190 | I | PX216473 | L | PX216491 |
| Saint Paul | PEN191 | A | PX216465 | AK | PX216516 |
| Saint Paul | PEN192 | K | PX216475 | I | PX216488 |
| Saint Paul | PEN193 | H | PX216472 | BF | PX216537 |
| Saint Paul | PEN194 | F | PX216470 | AQ | PX216522 |
| Saint Paul | PEN195 | H | PX216472 | W | PX216502 |
| Saint Paul | PEN196 | H | PX216472 | AP | PX216521 |
| Saint Paul | PEN197 | H | PX216472 | J | PX216489 |
| Saint Paul | PEN198 | H | PX216472 | AV | PX216527 |
| western Australia | PEN340 | G | PX216471 | AZ | PX216531 |
| South Africa | PEN341 | A | PX216465 | C | PX216482 |
| South Africa | PEN342 | A | PX216465 | E | PX216484 |
| South Africa | PEN343 | A | PX216465 | BN | PX216545 |
| South Africa | PEN344 | A | PX216465 | BG | PX216538 |
| South Africa | PEN345 | A | PX216465 | BG | PX216538 |
| South Africa | PEN346 | A | PX216465 | F | PX216485 |
| South Africa | PEN347 | A | PX216465 | BL | PX216543 |
| South Africa | PEN348 | A | PX216465 | BG | PX216538 |
| South Africa | PEN349 | A | PX216465 | A | PX216480 |
| South Africa | PEN350 | A | PX216465 | BO | PX216546 |
| South Africa | PEN351 | A | PX216465 | D | PX216483 |
| South Africa | PEN352 | A | PX216465 | BG | PX216538 |

**Supplementary Table 3.** Migration estimates performed within BayesAss v3.0.5 with 95% confidence intervals determined as the mean ± 1.96 x SD. Results are calculated between the two identified genetic clusters (K=2), and between all island populations in each ocean.

|  | | | **Migrants FROM** | | | | | | | | |
| --- | --- | --- | --- | --- | --- | --- | --- | --- | --- | --- | --- |
|  |  |  | **Atlantic Ocean** | | | | | **Indian Ocean** | | **K=2** | |
|  |  |  | Alex | Gough | Inaccessible | Nightingale | Tristan | Amsterdam | Saint Paul | Atlantic | Indian |
| **Migrants TO** | **Atlantic Ocean** | Alex | 0.6905(0.0222) | 0.0239(0.0222) | 0.0239(0.0223) | 0.0238(0.0220) | 0.1905(0.0425) |  | |  | |
|  |  | Gough | 0.0145(0.0140) | 0.7689(0.0316) | 0.0143(0.0138) | 0.0144(0.0139) | 0.1588(0.0342) |  |  |  |  |
|  |  | Inaccessible | 0.0161(0.0154) | 0.0160(0.0153) | 0.7612(0.0503) | 0.0192(0.0176) | 0.1559(0.0566) |  |  |  |  |
|  |  | Nightingale | 0.0119(0.0114) | 0.0118(0.0114) | 0.0119(0.0115) | 0.7178(0.0284) | 0.2228(0.0338) |  |  |  |  |
|  |  | Tristan | 0.0151(0.0145) | 0.0152(0.0146) | 0.0151(0.0144) | 0.0152(0.0144) | 0.9092(0.0308) |  |  |  |  |
|  | **Indian Ocean** | Amsterdam |  | | | | | 0.6804(0.0132) | 0.2539(0.0279) |  |  |
|  |  | Saint Paul |  |  |  |  |  | 0.0132(0.0127) | 0.9201(0.0279) |  |  |
|  | **K=2** | Atlantic |  | | | | | | | 0.9955(0.0044) | 0.0045(0.0044) |
|  |  | Indian |  |  |  |  |  |  |  | 0.0178(0.0121) | 0.9822(0.0121) |
